# Protective Effect of Photobiomodulation against Hydrogen Peroxide-Induced Oxidative Damage by Promoting Autophagy through Inhibition of PI3K/AKT/mTOR Pathway in MC3T3-E1 Cells

**DOI:** 10.1155/2022/7223353

**Published:** 2022-11-22

**Authors:** Xiaoshuang Zuo, Xinghui Wei, Cheng Ju, Xuankang Wang, Zhihao Zhang, Yangguang Ma, Zhijie Zhu, Xin Li, Zhiwen Song, Liang Luo, Xueyu Hu, Zhe Wang

**Affiliations:** ^1^Department of Orthopedics, Xijing Hospital, Fourth Military Medical University, Xi'an, Shaanxi, China; ^2^Department of Orthopedics, Tangdu Hospital, Fourth Military Medical University, Xi'an, Shaanxi, China; ^3^Hospital of People's Liberation Army Joint Logistic Support Force, Dalian, Liaoning, China

## Abstract

Photobiomodulation (PBM) has been repeatedly reported to play a major role in the regulation of osteoblast proliferation and mineralization. Autophagy is closely associated with various pathophysiological processes in osteoblasts, while its role in oxidative stress is even more critical. However, there is still no clear understanding of the mechanism of the role of autophagy in the regulation of osteoblast mineralization and apoptosis under oxidative stress by PBM. It was designed to investigate the impact of 808 nm PBM on autophagy and apoptosis in mouse preosteoblast MC3T3-E1 treated with hydrogen peroxide (H_2_O_2_) through PI3K/AKT/mTOR pathway. PBM could inhibit MC3T3-E1 cell apoptosis under oxidative stress and promote the expression of osteogenic proteins, while enhancing the level of autophagy. In contrast, 3-methyladenine (3-MA) inhibited the expression of osteoblast autophagy under oxidative stress conditions, increased apoptosis, and plus counteracted the effect of PBM on osteoblasts. We also found that PBM suppressed the activated PI3K/AKT/mTOR pathway during oxidative stress and induced autophagy in osteoblasts. PBM promoted autophagy of MC3T3 cells and was further blocked by 740 Y-P, which reversed the effect of PBM on MC3T3 cells with H_2_O_2_. In conclusion, PBM promotes autophagy and improves the level of osteogenesis under oxidative stress by inhibiting the PI3K/AKT/mTOR pathway. Our results can lay the foundation for the clinical usage of PBM in the treatment of osteoporosis.

## 1. Introduction

Osteoporosis, as a common systemic bone metabolism disease, is characterized by bone mass reduction and deterioration of bone structure resulting in enhanced bone fragility and increased fracture risk [1]. Osteoporosis adversely affects human health, especially in the aging populations. Globally, osteoporotic fractures occur approximately every 3 second, and nearly half of women and one-quarter of men over 50 years of age will suffer an osteoporotic fracture [2]. Hence, osteoporosis is a major threat to people's health and living standards. Therefore, effective treatment options for osteoporosis need to be established.

Oxidative stress is an important pathological process involved in osteoporosis [3]. During the development of oxidative stress, excessive reactive oxygen species (ROS) production destroys the balance between oxidation and antioxidant defense system, leading to osteoblast metabolic function disorder and apoptotic pathway activation [4, 5]. ROS, produced by oxidative stress, are the main intracellular signal transducers that maintain autophagy [6]. Moreover, a key role of autophagy is to regulate the levels of ROS [7, 8]. A few studies have indicated that regulating autophagy in cells under oxidative stress can effectively reduce osteoblast apoptosis and promote osteoblast differentiation, thus reducing bone loss [9, 10].

Photobiomodulation (PBM), a traditional physical therapy technique, has been extensively used in the treatment of skin disease, nerve injury, and other diseases owing to its anti-inflammatory and tissue repair-promoting effects and fewer side effects. Previous studies have shown that the main pathways of PBM include antiapoptosis, oxidative stress regulation, and autophagy [11–13]. However, Chang et al. revealed that PBM also enhance the proliferation and differentiation of osteoblasts and provided a new method to the regulation of osteoporosis and osteogenesis [14].

Thus, to explore the potential mechanism by which PBM regulates osteoporosis, an *in vitro* oxidative stress model was established, and the influence of PBM on H_2_O_2_ induced apoptosis and autophagy in MC3T3-E1 cells and bone formation was examined. We found that PBM regulated autophagy by affecting the PI3K/AKT/mTOR pathway, thus regulating osteoblast differentiation. The aim of this study was to investigate the potential of PBM in the regulation of osteoporosis. In addition to providing new treatment options for the prevention and cure of osteoporosis, these findings will also provide new avenues for research.

## 2. Materials and Methods

### 2.1. Cell Culture

The mouse MC3T3-E1 cells were obtained from the Type Culture Collection of the Chinese Academy of Sciences (Shanghai, China), which were cultivated in *α*-minimum essential medium (*α*-MEM; Hyclone, Cytiva, UK) added with 10% fetal bovine serum (Viva Cell, C04001-500, China) and penicillin-streptomycin double-resistant liquid (Solarbio, P1400, Beijing, China) in a humidified incubator with 5% CO_2_ at 37°C. There is an exchange of cell culture medium every three days. The cells were subcultured using 0.25% pancreatic protease containing ethylenediaminetetraacetic acid after reaching 80-90% confluence (Beyotime Biotechnology, C0201, Shanghai, China).

### 2.2. Cell Model Construction

This study was divided into the normal control group (no H_2_O_2_, no PBM); the H_2_O_2_ group (200, 300, 400, 500, 600 *μ*mol/L, 30% H_2_O_2_, Sinopharm Chemical Reagent Co., Ltd., China); the normal irradiation group (no H_2_O_2_, with PBM); the H_2_O_2_ irradiation group (with H_2_O_2_ and PBM); the antioxidant group (NAC, N-acetylcysteine, ST1546, Beyotime Biotechnology, China); the NAC irradiation group; the autophagic inhibition group (3-MA, 3-methyladenine, HY-1931, MedChemExpress, China); the 3-MA irradiation group; the PI3K agonist group (740 Y-P, TQ0003, TargetMol, USA); and the PI3K agonist irradiation group. All the inhibitor/agonist groups were pretreated with 2 mmol/L NAC, 5 mmol/L 3-MA, and 30 *μ*mol/L 740 Y-P for 2 h; next, the cells are treated with H_2_O_2_ for 4 h. The normal medium was replaced after 4 h incubation before photobiomodulation.

### 2.3. Irradiation Tools and Photobiomodulation

808 nm fiber-coupled laser device (MW-GX-808/1000 mW, near IR spectroscopy) for PBM treatment of MC3T3-E1 cells, manufactured by Changchun Leishi Optoelectronic Technology Co., Ltd. The parameters of photobiomodulation were as follows: 808 nm sequential wavelength, 6 mW/cm^2^ power density, 4.5 cm^2^ spot size, 17.01 J irradiated energy, 3.78 J/cm^2^ power density, and 630 s irradiation for one time per 24 h. The distance between the optical fiber and the bottom of the petri dish was about 21 cm. Equation of energy calculation formula is as follows: energy density (J/cm^2^) × spot size (cm^2^) = power (W) × time (s) (Table 1).

### 2.4. CCK-8

Activity of cells detected by Cell Counting kit-8 (C0005, Target Mol, USA). MC3T3 cells were irradiated with PBM at different time points (12, 24, 48, 72, 96h) and then washed with PBS. 10 *μ*L CCK-8 test solution was added to each well; for 3 h, the mixtures were placed in the dark at 37°C incubator. 450 nm absorbance was determined by a BioTek Synergy H1 microplate reader (BioTek, USA), which determined the survival rate of the cells.

### 2.5. ROS Assay

As directed by the manufacturer, the ROS content was detected by H2DCFDA kit (HY-D0940 MedChemExpress, China). The cells in each group were treated with different concentrations of H_2_O_2_ for 4 h, followed by irradiation with PBM. After 12 h, 5 *μ*m/L H2DCFDA working solution was used to detect ROS. The ROS fluorescence intensity was observed by a fluorescence microscope (ZEISS, Oberkochen, Germany) after incubation at 37°C for 30 min in darkness and washed three times with PBS.

### 2.6. Apoptosis Assay

Apoptosis was analysis by flow cytometry (BD Accuri® C6, BD Biosciences). The induction of cell apoptosis after H_2_O_2_ and PBM treatment was verified using the apoptosis detection kit (BD, 556547, Pharmingen, America). Fluorescence signals of FITC and PI staining cells were collected by flow cytometry, and BD Accuri™ C6 flow cytometry system analyzed the outcomes.

### 2.7. Transmission Electron Microscopy (TEM)

MC3T3 cells were pretreated with H_2_O_2_ for 4 h and then irradiated with PBM. The cells were fixed by 2.5% glutaraldehyde and 1% osmium. The samples were dehydrated by ethanol gradient and dried by epoxy propane to prepare ultrathin sections. Then, transmission electron microscopy (JEM-1400 flash) was used to capture images of autophagosomes from fixed cell sections at 10000x magnification.

### 2.8. Western Blotting Assay

Total protein in the cells were extracted with100 *μ*L RIPA (P0013B, Beyotime Biotechnology, China) and centrifuged at 12,000 rpm for 15 min. Protein concentrations were determined using the BCA protein kit (Thermo Scientific, 23227). Protein denaturation was performed with 5x SDS-PAGE protein loading buffer (P0015, Beyotime Biotechnology). 8%, 10%, or 12% SDS-PAGE gels to isolate proteins and nitrocellulose membrane were used for the transfer of proteins (P-N66485, Pall, America). The membrane was sealed with 5% skim milk powder for 2 h and then incubated at 4°C with the primary antibodies as follows: osterix (OST, TD7731, Abmart, 1 : 1000); RUNX2 (PA0631, Antiprotech, 1 : 1000); osteocalcin (OCN, GTX55255, GeneTex, 1 : 1000); osteopontin (OPN, PA5772, Antiprotech, 1 : 1000); microtubule-associated protein 1A/1B-light chain 3 (LC3B, T55992, Abmart, 1 : 1000); p62 (T59081, Abmart, 1 : 1000); Bcl2 (GTX100064, GeneTex, 1 : 1000); Bax (14-6997-82, Invitrogen, 1 : 1000); PI3K (60225-1-IG, Proteintech, 1 : 1000); p-PI3K (AF3241, Affinity, 1 : 1000); AKT (4691, Cell Signaling Technology, 1 : 1000); p-AKT (4060, Cell Signaling Technology, 1 : 1000); mTOR (ab134903, Abcam, 1 : 1000); p-mTOR (ab109268, Abcam, 1 : 1000); *β*-actin (66009-1-IG, Proteintech, 1 : 2000); and GAPDH (60004-1-IG, Proteintech, 1 : 3000). The strips are treated with 1x TBST, afterward, followed by relative secondary antibodies for 1 h (goat antirabbit (H+L) HRP (SA-10011); goat antimouse (H+L) HRP, SA-10010, InCellGene, TX, USA, 1 : 3000). An ultrasensitive luminescent solution is then used to detect proteins on the membrane. Amersham Imager 600 (general electric) was used to observe the bands, and the protein band densities were analyzed by the Image J V1.6 software.

### 2.9. Transcriptional Polymerase Chain Reaction

RNA was extracted from cells using TRIzol reagent (15596026,Invitrogen™, USA) then, use Evo M-MLV RT Premix to reverse transcribe RNA to cDNA for RT-PCR (AG11706, Accurate Biotechnology, Hunan). The reaction conditions were 37°C 15 min, 85°C 5 s, and 4°C 10 min by real-time fluorescent quantitative PCR kits (AG11701, Accurate Biology, Hunan), and real-time fluorescence quantitative reaction system includes 1 *μ*L cDNA, 1 *μ*L primers, 5 *μ*L SYBR Green, and 3 *μ*L RNase/DNase-free water; general volume was 10 *μ*L. The primers were sequenced as shown in Table 2. *β*-Actin is a housekeeping gene, and the data were computed by 2^−△△CT^ method.

### 2.10. Immunofluorescence

After 48 h of treatment, four percent paraformaldehyde was used for fixation of cells. Cells were then treated with 0.3% Anapoe-X-100 for 30 min, blocked with 5% BSA for 1 h, and treated with LC3 antibody (T55992, Abmart, 1 : 200). The cells were incubated with fluorescent secondary antibody (Alexa Fluor 488-Affinipure Goat anti-rabbit IgG (h + L), Jackson, America) for 40 min overnight. Counterstaining with DAPI (C0065, Solarbio, China) was used to highlight nuclei. Fluorescence expression of the stain was observed with a ZEISS fluorescence microscope (Axio Observer, Germany), and the speck photos under different field views were randomly selected and analyzed by using objective lens. Image-Pro Plus software (V6.0) was used to count the number of specks.

### 2.11. Alkaline Phosphatase Staining

Alkaline phosphatase (ALP) kit (C3206, Beyotime Biotech, China) was used to detect the expression of ALP. Place 5 × 10^4^ cells/well in 12-well culture dishes; after adherence, H_2_O_2_ was used to induce cell damage for 4 h, and then, the cells were treated with PBM. To promote osteoblast mineralization, osteogenic medium was used to induce the cells for 7 days (changed culture medium every 2 days). Osteogenic induction medium was prepared as follows: add 50 *μ*g/mL ascorbic acid, 10 mM B-glycerophosphate, and 10 nmol dexamethasone (795437, G9422, D4902, Sigma-Aldrich, St. Louis, MO, USA) in normal *α*-MEM complete medium. After washing with PBS, the cells were fixed and stained in accordance with the kit instructions. The samples were stained with BCIP/NBT substrate for 30 min in the dark. Lastly, after washing twice with distilled deionized H_2_O, we observed and photographed the samples with light microscope.

### 2.12. Alizarin Bordeaux Staining

Extracellular matrix mineralization was assayed with an alizarin red S (C0148S, Beyotime Biotechnology, China). Seed 5 × 10^4^ cells/well in 6-well culture dishes. After cell apposition and dissemination for 24 h, H_2_O_2_ treatment was performed by 200 *μ*m H_2_O_2_ treatment for 4 h; then, PBM irradiation was performed for 630 s. After 48 h, cells were cultured for 21 d using osteogenic induction medium; and the medium was changed every 3 d. The cells were rinsed in PBS without calcium and magnesium after medium removal. The cells were fixed in fixative for 20 min and washed in PBS three times. The cells were stained with alizarin red stain solution for 30 min. The last step was to wash the cells using deionized water; next, all cells were photographed with microscope.

### 2.13. Statistical Analysis

All the experimental results in this experiment are data obtained in at least 3 independent repetitions. ImageJ (V1.47, National Institutes of Health, USA) software helps to analyze immunofluorescence data of fluorescence intensity and the gray scale of WB bands. LC3 immunofluorescence results were performed using ImageJ Pro Plus software (V6.0, Media Cybernetics, USA). The statistical analyses and line graph and histogram in the results were conducted and drawn using GraphPad Prism (V9.0 GraphPad Software, La Jolla, CA) software. All data are expressed as mean ± standard deviation (SD). Unpaired *t*-test was used for comparison between the two groups. One-way analysis of variance was used to determine differences between three or more groups, and Tukey's multiple comparison test was performed if statistically significant. *p* value < 0.05 was considered as statistically significant difference.

## 3. Result

### 3.1. PBM Enhanced the Activity of MC3T3 Cells under Oxidative Stress

**Figure 1:**
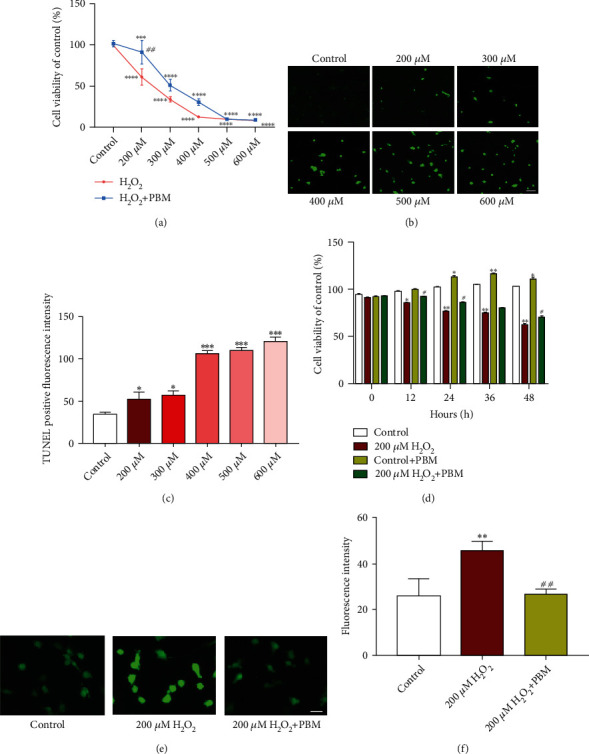
PBM enhanced the cellular activity of MC3T3-E1 cells under oxidative stress. (a) CCK-8 assay for cell survival. Relative cell survival of MC3T3 cells cultured with different concentrations of H_2_O_2_ (*n* = 3). (b, c) The apoptosis was measured by TUNEL assays. Quantification of TUNEL-positive cells in every group (*n* = 3). White scale bar = 200 *μ*m. (d) Total ROS were determined using DCFH-DA. MC3T3-E1 cells were cultured with 200 *μ*mol/L H_2_O_2_ and treated with PBM for different time. Compared with the H_2_O_2_ group, the number of viable cells was significantly increased (*n* = 3). (e, f) PBM treatment with 200 *μ*m hydrogen peroxide induced MC3T3-E1 cells. ROS fluorescence intensity decreased significantly. Quantification of the amounts of positive cells expressing ROS in per group (*n* = 3). White scale bar = 100 *μ*m. The *p* value < 0.05 significance level was considered significant. ^∗^mean vs. control. ^#^mean vs. 200 *μ*mol/L.

Oxidative stress has been shown to induce apoptosis in a variety of cells [15–17]. Initially, using different concentrations of H_2_O_2_ to treat MC3T3 cells (200, 300, 400, 500, and 600 *μ*mol/L) for 4 h [18]. Cell Counting Kit was used to detect absorbance values, calculate cell viability, and observe the effect of different concentrations of hydrogen peroxide on the proliferation of MC3T3 cells. According to Figure 1(a), the viability of MC3T3 cells gradually diminished with the enhancement in H_2_O_2_ concentration vs. the control group. Our results showed a dose-dependent inhibitory effect (*p* < 0.001). With the exception of the group of 200 *μ*mol/L H_2_O_2_, there was a significant decrease in cell viability in all groups of H_2_O_2_ compared to the control group. After PBM irradiation, the survival rate of the PBM+H_2_O_2_ group was notably lower vs. control group (*p* < 0.001), where the cell activity of the 200 *μ*mol/L H_2_O_2_ group was close to 60%-70% than that of the control group. Furthermore, all PBM+H_2_O_2_ groups was increased vigorously compared with H_2_O_2_ group, with the 200 *μ*mol/L H_2_O_2_ group showing a significant improvement in cell activity after PBM irradiation (*p* < 0.05), and PBM can promote cell survival with H_2_O_2_ condition. At the same time, the degree of apoptosis osteoblasts exposed to different concentrations of hydrogen peroxide was using the TUNEL assay (Figures 1(b) and 1(c)). Within the concentration range we selected, the apoptosis of osteoblasts was positively proportional to the concentration of hydrogen peroxide. Based on the above results, 200 *μ*mol/L H_2_O_2_ was selected to establish an osteoblast oxidative damage model in MC3T3 cells. MC3T3 cells treated with 200 *μ*mol/L hydrogen peroxide were irradiated by PBM.

The multiplication of MC3T3 cells was detected by CCK-8 at different time points (0, 12, 24, 36, and 48 h) after irradiation. The results revealed that MC3T3 cells were significantly enhanced after 24 h of PBM (*p* < 0.05). The activity of MC3T3 cells treated with 200 *μ*mol/L hydrogen peroxide was time-dependently reduced, and the activities of MC3T3 cells were restored after PBM and significantly increased after 24 h (*p* < 0.01), suggesting that PBM can inhibit the oxidative damage caused by hydrogen peroxide to MC3T3 cells and improve the survival rate of MC3T3 cells (Figure 1(d)). To examine whether the influence of PBM on cells correlates with its antioxidant properties, we used the fluorescent probe DCFH-DA to generate ROS. The data from Figures 1(e) and 1(f) shows that ROS expression was clearly increased after treatment of MC3T3 cells with H_2_O_2_. However, when the cells were irradiated with PBM, the expression of ROS was reduced, and the outcomes revealed that PBM attenuated the level of oxidative stress in MC3T3 cells.

### 3.2. PBM Inhibited H_2_O_2_-Induced Apoptosis of MC3T3 Cells and Promoted Osteogenic Differentiation

**Figure 2:**
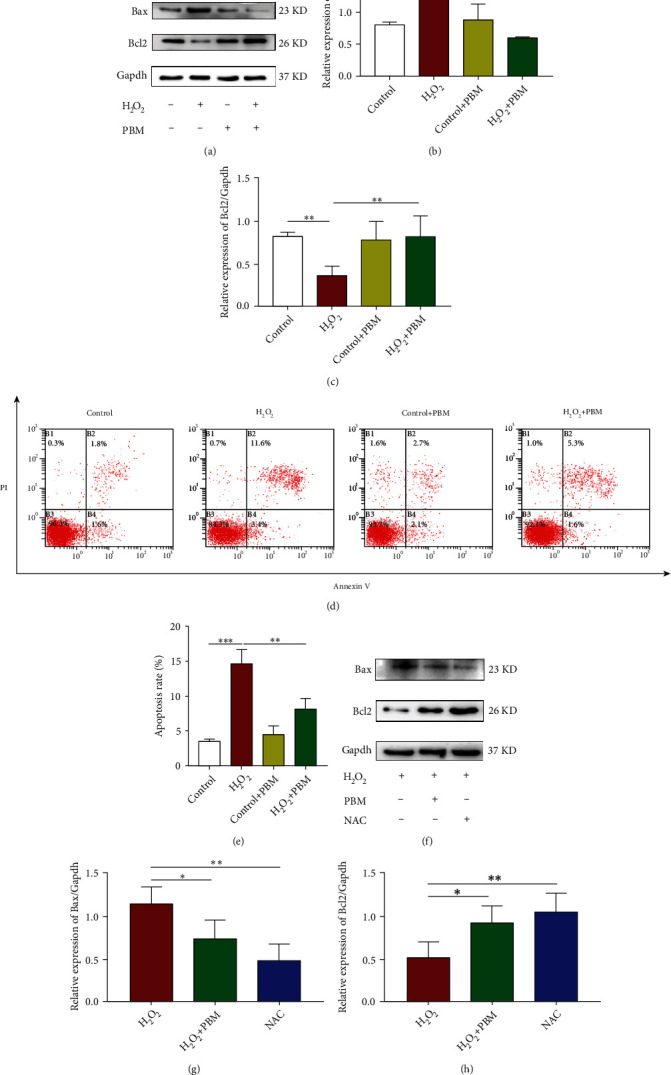
PBM inhibited H_2_O_2_-induced apoptosis of MC3T3 cells. (a–c) Western blotting was performed to test the Bax and Bcl2 levels in the control, H_2_O_2_, and treated with PBM groups (*n* = 4 individuals per group). (d, e) Cell apoptosis assays via flow cytometry. The proportions of prematurely apoptotic cells were increased by H_2_O_2_ treatment, but PBM can mitigate this influence. (f–h) Western blotting analysis of Bax and Bcl2 levels in the H_2_O_2_, H_2_O_2_+PBM, and NAC group (*n* = 4). A *p* value of 0.05 or 0.01 should be considered.

The effect of PBM on H_2_O_2_-induced Bcl-2 and Bax expression in MC3T3 cells was detected by western blot. Bax expression level was significantly upregulated, and Bcl-2 protein expression level was downregulated after H_2_O_2_ treatment in MC3T3 cells. After irradiation with PBM, enhanced Bcl-2 expression and decreased expression were observed. Besides, the apoptosis of cells irradiated by PBM has been significantly inhibited (Figures 2(a)–2(c)). Similarly, flow cytometry results confirmed an increased apoptosis rate in MC3T3 cells treated with 200 *μ*mol/L hydrogen peroxide. The apoptosis rate of MC3T3 cells irradiated with PBM did not show appreciable changes. However, the apoptotic rate of H_2_O_2_-stimulated MC3T3 cells was significantly reduced with PBM treatment (Figures 2(d) and 2(e)). As a positive control, the ROS scavenger N-acetylcysteine (NAC) significantly reduced oxidative stress levels after 2 h of cell pretreatment (Figures 2(f)–2(h)). These achievements suggest that PBM attenuates H_2_O_2_-induced apoptosis in MC3T3 cells.

**Figure 3:**
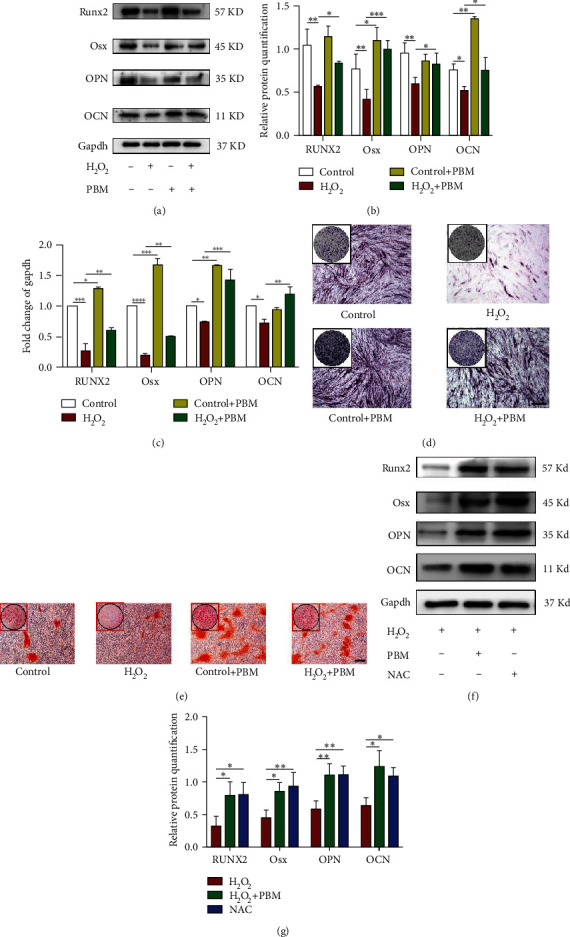
PBM promoted MC3T3 cell osteogenic differentiation. (a, b) An analysis of Runx2, Osx, OPN, and OCN expressions by western blotting in the control, H_2_O_2_, and treated with PBM groups (*n* = 4 individuals per group). (c) RT-PCR was applied to measure the levels of Runx2, Osx, OPN, and OCN mRNA in each group. (d) Alkaline phosphatase dyeing. (e) Alizarin red dyeing; black scale bar shows 200 *μ*m. (f, g) An analysis of Runx2, Osx, OPN, and OCN expressions by western blotting in the H_2_O_2_, H_2_O_2_+PBM, and NAC groups (*n* = 4). The *p* value < 0.05 significance level was considered significant.

The expression of osteogenic-related proteins RUNX2, Osterix, OPN and OCN was detected by Weatern blotting method. Also, the degree of cell differentiation and mineralization was observed using ALP and alizarin red staining to investigate the influence of PBM on H_2_O_2_-induced osteoblast dysfunction. The presence of H_2_O_2_ significantly reduced the expression of osteogenic-related proteins in cells compared to controls, and PBM significantly restored the expression of these proteins inhibited by H_2_O_2_ (Figures 3(a) and 3(b)). The RT-PCR outcomes are presented in Figure 3(c); the mRNA levels of RUNX2, osterix, OPN, and OCN were clearly reduced in the H_2_O_2_ group as compared to the control group. The mRNA expression levels of RUNX2, osterix, OPN, and OCN were increased in the H_2_O_2_-PBM group with respect to the H_2_O_2_ group. To confirm the level of osteoblast differentiation and mineralization in the presence of PBM with H_2_O_2_, ALP staining and alizarin red staining were performed. ALP is an indicator of osteoblast differentiation cells irradiated by PBM and subsequently cultured with osteogenic induction solution. At 14 days, ALP expression was reduced in MC3T3 cells from H_2_O_2_-treated cells compared to the control group. The ALP staining intensity of PBM-irradiated MC3T3 cells slightly increased. Compared to that of the H_2_O_2_ group, the ALP staining intensity of the H_2_O_2_ group after PBM irradiation was significantly increased (Figure 3(d)). Alizarin red staining was used to measure osteoblast differentiation twenty-one days after induction of cells with osteogenic induction solution, what the results showed is that calcium deposition decreased in the H_2_O_2_ group compared with that in the control group; the level of calcium deposition was slightly increased in the PBM-irradiated MC3T3 cells. After PBM, calcification was significantly greater in the H_2_O_2_ group than that in the control set (Figure 3(e)). According to these results, PBM promoted osteogenic differentiation of MC3T3 preosteoblasts.

For further studies on osteoblast differentiation influenced by PBM with oxidative stress, MC3T3 cells were pretreated with NAC (2 mmol/L) and then cultured with H_2_O_2_. In the western blotting experiment, we also observed an elevated expression of osteogenic proteins in cells exposed to the antioxidant NAC, and this effect was more pronounced after PBM (Figures 3(f) and 3(g)). Taken together, these results proved that PBM can reduce apoptosis triggered by oxidative stress and promote osteogenic differentiation in MC3T3 cells, and the combined effect of PBM with antioxidant NAC is more significant.

### 3.3. PBM Enhanced the Autophagy Level Induced by H_2_O_2_ in MC3T3 Cells

**Figure 4:**
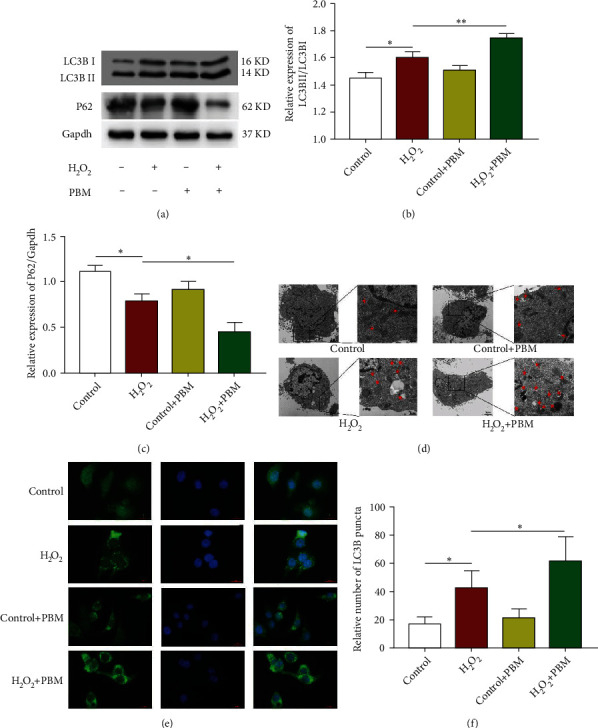
PBM enhanced the autophagy level induced by H_2_O_2_ in MC3T3 cells. (a–c) Western blot detection of LC3B and P62 expression levels in the control, H2O2, and PBM-treated groups (*n* = 4 individuals per group). (d) TEM was used to identify autophagosomes in MC3T3 cell treated with H_2_O_2_ and PBM, scale bar: 2 *μ*m and 500 nm. (e, f) LC3B-positive punctate structures were determined by immunofluorescence (*n* = 3 individuals per group). Scale bar = 20 *μ*m; *p* values < 0.05 or 0.01.

Since autophagy is proposed to hold an important role in osteoblast differentiation, we first studied the change in autophagy levels of H_2_O_2_-induced MC3T3 cells after PBM treatment (Figure 4(a)).

LC3B is an essential marker of macroautophagy. We detected autophagy in MC3T3 cells by western blotting, and based on the literature, it is also clear from our results that the autophagy level of normal MC3T3 cells increased after PBM [19]; however, the difference was not found to be statistically meaningful (*p* > 0.05).

The autophagy level of MC3T3 cells induced by H_2_O_2_ is shown in Figure 4(b); in comparison with the controlling group, LC3B-II/LC3B-I ratio was enhanced and p62 protein level was reduced in the H_2_O_2_ group (*p* < 0.05), LC3B-II/LC3B-I ratio was further increased, and p62 expression was significantly inhibited after PBM (*p* < 0.05) (Figure 4(c)). The cellular ultrastructure was viewed by transmission electron microscopy (TEM), and this approach is considered to be the standard for detecting autophagy. Ultrastructure and number of autophagic vesicles in osteoblasts under oxidative stress were observed by TEM (Figure 4(d)). As anticipated, autophagosomes were enclosed by a double-membrane structure in the cells. The autophagic vesicles of MC3T3 cells were slightly increased after PBM irradiation comparing with the control group, but it was not statistically significant. The number of H_2_O_2_-induced autophagosomes in MC3T3 cells was significantly increased after PBM compared with the H_2_O_2_ group. Besides, H_2_O_2_ enhanced the accumulation of LC3B puncta in the nucleus; however, this phenomenon was further enhanced after PBM (*p* < 0.05) (Figures 4(e) and 4(f)). Such results suggested that H_2_O_2_ can induce cellular autophagy and that PBM may further promote autophagy by activating the self-repair of cells under oxidative stress to scavenge excessive ROS in cells, thus promoting osteoblast differentiation.

### 3.4. Autophagy Inhibitors Can Counteract the Effect of PBM on H_2_O_2_-Induced MC3T3 Cells

**Figure 5:**
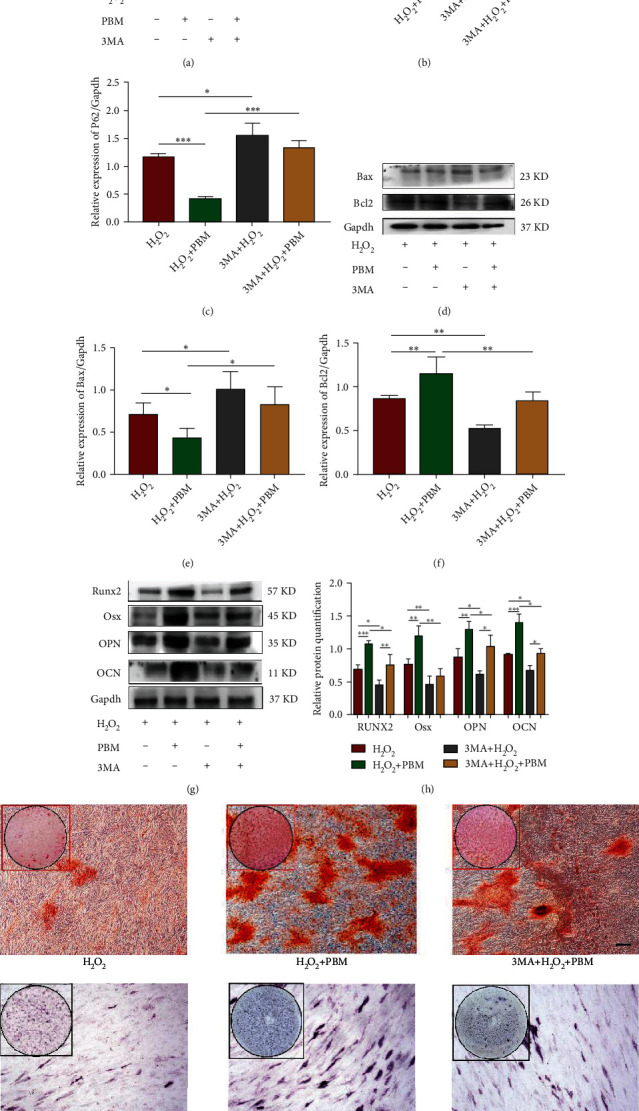
Autophagy inhibitors can reverse the impact of PBM on H_2_O_2_-induced MC3T3 cells. (a–c) Western blotting assays of the LC3B and P62 expression levels of in the H_2_O_2_, 3-MA, and treated with PBM groups (*n* = 4). (d–f) The levels of Bax and Bcl2 expressions in the H_2_O_2_, 3-MA, and treated with PBM groups (*n* = 4) were measured by western blotting. (g, h) Western blotting was performed to assay the levels of Runx2, Osx, OPN, and OCN in the H_2_O_2_, 3-MA, and treated with PBM groups (*n* = 4). (i) Alphabetic staining and alizarin red staining of MC3T3 cells processed with H_2_O_2_, 3-MA, and PBM. The *p* values < 0.05, 0.01, and 0.001 and the scale bar represents 200 *μ*m.

To further confirm whether the effect of PBM on MC3T3 cell differentiation was dependent on an increase in autophagy, the autophagy inhibitor 3-MA was used. Detection of expression of autophagy-related proteins LC3B and p62 by western blot (Figures 5(a)–5(c)), and the results revealed that the presence of the autophagy inhibitor 3-MA significantly inhibited autophagy induced by H_2_O_2_ in MC3T3 cells. Apoptosis-related proteins Bax and Bcl2 were measured by western blotting. Figures 5(d)–5(f) show that the presence of the autophagy inhibitor 3-MA significantly promoted the apoptosis of MC3T3 cells due to H_2_O_2_. Figures 5(g) and 5(h) show the osteogenic-related proteins Runx2, osterix, OCN, and OPN; the results indicated that the presence of 3-MA significantly reduced the capacity of MC3T3 cells for osteogenic differentiation.

However, after PBM irradiation, autophagy and apoptosis levels were not significantly changed, but osteogenic-related proteins were significantly increased; ALP and alizarin red staining results also showed more clearly that PBM-irradiated MC3T3 cells induced by 3-MA and H_2_O_2_; the calcification level of MC3T3 cells was lower than that of H_2_O_2_+PBM group (Figure 5(i)). Collectively, these results suggest that 3-MA counteracts the influence of PBM on H_2_O_2_-induced cells and confirm that PBM inhibited apoptosis of MC3T3 cells and enhanced osteogenic differentiation ability by promoting autophagy.

### 3.5. PI3K/AKT/mTOR Pathway Is Engaged in the Promotion of H_2_O_2_-Induced Autophagy in MC3T3 Cells by PBM

**Figure 6:**
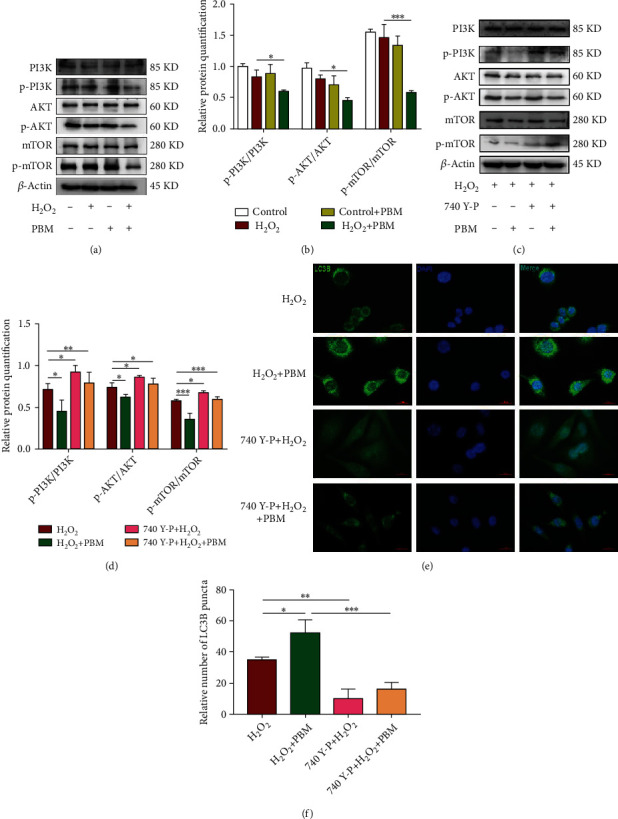
Participation of PI3K/AKT/mTOR pathway in PBM to promote H_2_O_2_-induced autophagy in MC3T3 cells. (a, b) Levels of p-PI3K, p-AKT, and p-mTOR exposure in the control, H_2_O_2_, and PBM-treated groups were detected by western blotting (*n* = 4). (c, d) Western blotting analysis and quantification of the levels of the p-PI3K, p-AKT, p-mTOR in H_2_O_2_, H_2_O_2_+PBM, and 740 Y-P+PBM group (*n* = 4). (e, f) LC3B-positive punctate structures were determined by immunofluorescence. The number of positive LC3B cells in the H_2_O_2_+PBM and 740 Y-P+PBM groups was calculated (*n* = 3). The scale bar shows 20 *μ*m; a statistically significant difference is defined as ^∗^*p* < 0.05.

The PI3K/AKT/mTOR pathway is essential to activation of autophagy. To investigate the effects of PI3K/AKT/mTOR pathway in H_2_O_2_-induced regulation of PBM in MC3T3 cells, western blotting was used to test the activation of this pathway (Figure 6(a)). As shown in Figure 6(b), the expression levels of p-PI3K/PI3K, p-Akt/AKT, and p-mTOR/mTOR were slightly reduced in the H_2_O_2_ group compared with the controlling group. But without statistical significance (*p* > 0.05), there are significantly lower expression levels of p-PI3K, p-Akt, and p-mTOR in the H_2_O_2_+PBM group than in the H_2_O_2_ group (*p* < 0.05). In conclusion, these results suggest that PBM may enhance autophagy through inhibiting the PI3K/Akt/mTOR pathway.

To validate whether PBM promotes autophagy in MC3T3 cells through the PI3K/AKT/mTOR pathway, we examined changes in the pathway and autophagy after treatment with the PI3K agonist 740 Y-P and PBM. As illustrated in Figures 6(c) and 6(d), western blotting results showed the protein expression levels of p-PI3K, p-Akt, and p-mTOR in the different groups. The expression levels of p-PI3K/PI3K, p-Akt/AKT, and p-mTOR/mTOR proteins were significantly increased in the cells after PI3K agonist treatment compared with the H2O2 group. The expression levels of p-PI3K/PI3K, p-Akt/AKT, and p-mTOR/mTOR were significantly decreased after PBM irradiation compared with the PI3K agonist group. As shown in Figures 6(e) and 6(f), the immunofluorescence results showed that the green fluorescent scattered spots around the nucleus were dramatically reduced, and the autophagy protein LC3B was reduced, which verified that the PI3K receptor agonist 740 Y-P inhibited autophagy by promoting PI3K. After PBM, the green fluorescent spots around the nucleus increased significantly, which could significantly promote the autophagy level of MC3T3 cells under H_2_O_2_ conditions. These outcomes illustrated that 740Y-P decreased LC3B expression and inhibited autophagy in MC3T3 cells under H_2_O_2_ condition through activating the PI3K/AKT/mTOR pathway. PBM then enhanced cellular autophagy by inhibiting activation of the PI3K/AKT/mTOR pathway in MC3T3 cells with H_2_O_2_.

## 4. Discussion

Oxidative stress occupies the core site in the pathological process of osteoporosis, and osteoporosis caused by excessive oxidative stress may be a key factor affecting its prognosis [20, 21]. Oxidative stress can cause a series of alterations in cells and activate the apoptotic signaling pathway, leading to cellular dysfunction [22]. The excessive production of ROS is part of the major pathophysiological basis of oxidative stress. Being a product of normal redox reactions *in vivo*, ROS can participate in the modulation of bactericidal, detoxification, and multiple metabolic pathways under normal physiological conditions [23]. However, oxidative stress caused by the excessive production of ROS, which damages multiple components of osteoblasts and inhibits osteoblast proliferation, differentiation, and bone formation, has been shown to be a vital factor affecting the development of osteoporosis [24–26]. In the previous study, we choose H_2_O_2_ as an effective oxidant to induce oxidative stress, which could induce excess ROS produced by the organism to penetrate the cell membrane and diffuse rapidly intracellularly, thereby mimicking the pathophysiological process of oxidative stress [23, 27].

Photobiomodulation therapy, also known as weak low-laser-level therapy [28], which is noninvasive, anti-inflammatory, and prorepair, has been widely used in clinical practice. With the gradual exploration of PBM, many researchers have investigated the role of PBM in osteoblasts and found that PBM can significantly facilitate multiplication of MC3T3-E1 cells [29]. De Marco et al. suggested that PBM could improve fracture healing in rats and promote bone regeneration in bone defects [30]. Other studies have suggested that different wavelengths of PBM have significant regulatory effects on osteoblast differentiation [14]. We had previously applied PBM to the therapy of spinal cord injury and found that PBM could reduce the inflammatory response, reduce oxidative stress, inhibit neuronal apoptosis, and promote repair of spinal cord injury functions [31–33]. Although oxidative stress is one of the main inhibitors of osteoblast differentiation, PBM regulates osteoblast proliferation. In particular, the mechanisms of that regulate osteoblast differentiation during oxidative stress are not clear. Therefore, we investigated the regulatory mechanism of PBM in osteoblast differentiation under oxidative stress.

In this study, we constructed an osteoblast oxidative damage model using MC3T3-E1 cells cultured with the appropriate concentration of H_2_O_2_ (200 *μ*mol/L) *in vitro*. To confirm the effect of hydrogen peroxide on the oxidative damage of osteoblasts, we evaluated the activity of osteoblasts using CCK-8. The apoptosis rate of osteoblasts was measured using flow cytometry, and the oxidative stress level was measured using an ROS probe. With PBM treatment, H_2_O_2_-induced MC3T3-E1 cell activity increased and the intensity of ROS florescence became weak, indicating that PBM has an antiapoptotic effect on the survival of oxidized osteoblasts. The findings supported that PBM may act as an antioxidant to regulate the oxidative stress levels of osteoblasts.

Autophagy is one of major mechanisms that promotes cell viability. It functions as a regulator in intracellular homeostasis mainly through the degradation and recycling of intracellular metabolites. In stressful microenvironments such as oxidative stress, hypoxia, and nutrient deprivation conditions, autophagy is activated, which in turn inhibits the accumulation of ROS, thus exerting an inhibitory effect on apoptosis and reducing oxidative stress [34, 35]. At the same time, researchers discovered that autophagy has a major role in maintaining bone homeostasis and osteoblast differentiation [36]. Intracellular mineralized crystals can be transported by autophagosomes to promote extracellular mineralization [37]. In contrast, after the inhibition of autophagy, *in vitro* studies showed that the degree of cell mineralization was reduced, and bone mass and volume *in vivo* were also reduced, ultimately leading to oxidative stress [38]. Bostanciklioglu et al. used different laser irradiation wavelengths in a rat model of mucositis and found that autophagy was activated [39]. These results demonstrate not only that autophagy has a critical and central role in osteogenesis and differentiation but also that PBM plays a regulatory role in autophagy.

TEM of autophagic vesicles and other subcellular structures in a double-layer membrane-like structure is the gold standard for detecting autophagy [40]. However, it can only reflect the presence or absence of autophagy and not the level of autophagic activity. Among the numerous studies on autophagy, the ratio of LC3-II/LC3-I is a classical marker of autophagy, which reflects the strength of autophagic activity, whereas the degradation of p62 is an important marker of autophagy [41, 42]. Accordingly, we detected the expression of autophagy marker proteins LC3-I, LC3-II, and p62 using western blotting to reflect the impact of H_2_O_2_ on osteoblast autophagy and the therapeutic effect of PBM.

In the present study, LC3 expression increased after H_2_O_2_ induction in MC3T3 cells, suggesting that oxidative damage to MC3T3 cells by H_2_O_2_ stimulated osteoblast autophagy. This discovery is in line with the role indicated by She et al. in their study on enhanced autophagy of osteoblastoma MG63 cells under oxidative stress conditions [43]. We considered that after the oxidative stress occurs, large amounts of ROS were generated, and apoptosis was promoted. Nevertheless, apoptosis is accompanied by the activation of autophagy to remove damaged organelles in a timely manner. This phenomenon is a self-help behavior of cells, but their ability is limited. After PBM treatment, we found a further rise in LC3 expression, which indicates a further enhancement of autophagy levels in MC3T3 and a decrease in intracellular oxidative stress. We found that PBM can effectively remove damaged fragments from osteoblasts by inducing autophagy, reducing oxidative stress, and inhibiting apoptosis. Therefore, we speculate that a further increase in autophagy after PBM irradiation of osteoblasts under oxidative stress conditions could be an important way to protect cells from the antioxidant stress response.

Investigating the role of autophagy on osteoblasts under oxidative stress conditions and in protecting osteoblasts from PBM-induced oxidative damage, we used 3-MA to inhibit autophagy in osteoblasts in our research, and our results suggested that suppression of autophagy after H_2_O_2_-induced oxidative damage in osteoblasts resulted in increased levels of oxidative stress and reduced osteogenic differentiation of osteoblasts. The protection of PBM on osteoblasts is invalid after the inhibition of autophagy, which is consistent with the protective effect of autophagy on osteoblasts damaged be oxidation reported by Shi et al. and Yang et al. [44, 45]. Hence, PBM may defend osteoblasts from oxidative damage by regulating the level of autophagy in damaged osteoblasts. In summary, we demonstrated that PBM reduced oxidative stress, promoted osteoblast survival and differentiation, and attenuated the effects of H_2_O_2_ on osteoblast apoptosis. We also found that during this process, PBM promoted the level of autophagy in osteoblasts under oxidative stress, increased the expression of autophagy-related proteins LC3-II and LC3-I, and diminished the expression of p62, while 3-MA reversed the regulation of oxidative stress levels by PBM. This indicated that the antioxidant protective effect of PBM on osteoblasts damaged by oxidative stress is closely related to autophagy. Therefore, we propose that PBM may reduce oxidative stress levels through inducing autophagy to inhibit the influence of H_2_O_2_ in osteoblast apoptosis and promote osteoblast survival and differentiation. However, the mechanism by which PBM induces autophagy and the pathways by which PBM activates to promote autophagy remain unclear.

It is a common knowledge that the PI3K/AKT/mTOR pathway is engaged in various cellular physiological and pathological processes, including cell growth, apoptosis, and autophagy [46, 47]. Suppression of PI3K/AKT/mTOR pathway may activate autophagy [48, 49]. Thus, we examined the impact of PBM on PI3K/AKT/mTOR osteoblasts under oxidative stress. As an intracellular phosphatidylinositol kinase, PI3K has a serine/threonine kinase activity. Phosphorylated PI3K can produce a second messenger phosphatidylinositol-3,4,5-triphospholipid (PIP3) on the cell membrane, which binds to the intracellular signaling proteins AKT and phosphoinositol-dependent enzymes (PDK1). AKT activation is caused by phosphorylation of the threonine and serine sites of AKT [50, 51]. As a negative regulator of autophagy initiation, mTOR blocks ATG1-activated kinase [52]. H_2_O_2_ induced oxidative stress in osteoblasts after PBM, and PI3K/AKT/mTOR protein phosphorylation levels were detected. The findings revealed that the expression of p-PI3K, p-AKT, and p-mTOR was slightly decreased under oxidative stress conditions. PI3K/AKT phosphorylation levels were significantly reduced and LC3 expression levels were enhanced after PBM irradiation, suggesting that PBM inhibits the activation of PI3K/AKT/mTOR pathway by downregulating PI3K/AKT/mTOR phosphorylation, thereby promoting autophagy. To investigate further the inhibitory role of PBM on this pathway, we employed 740 Y-P, PI3K agonist, and revealed that 740Y-P markedly upregulated the expression of PI3K/AKT/mTOR phosphorylated protein in MC3T3 cells and facilitated PI3K/AKT/mTOR pathway activation. When 740 Y-P was used in combination with PBM, the promotion of autophagy by PBM was no longer significant, indicating that the inhibitory action of PBM on the PI3K/AKT/mTOR pathway was counteracted by 740 Y-P.

Our present study has some limitations. Because our existing optical fiber used for *in vivo* research can only be used for local irradiation and cannot meet the requirements of whole-body irradiation of animals, we are improving the optical fiber to be more suitable for PBM irradiation for osteoporosis treatment *in vivo*. Therefore, we have not yet carried out verification *in vivo*, but this research will be conducted in the future. The regulation of autophagy is complex, and we only confirmed the impact of PBM on this pathway. However, the direct target of PBM on osteoblasts and how PBM inhibits phosphorylation need to be further studied.

## 5. Conclusion

**Figure 7:**
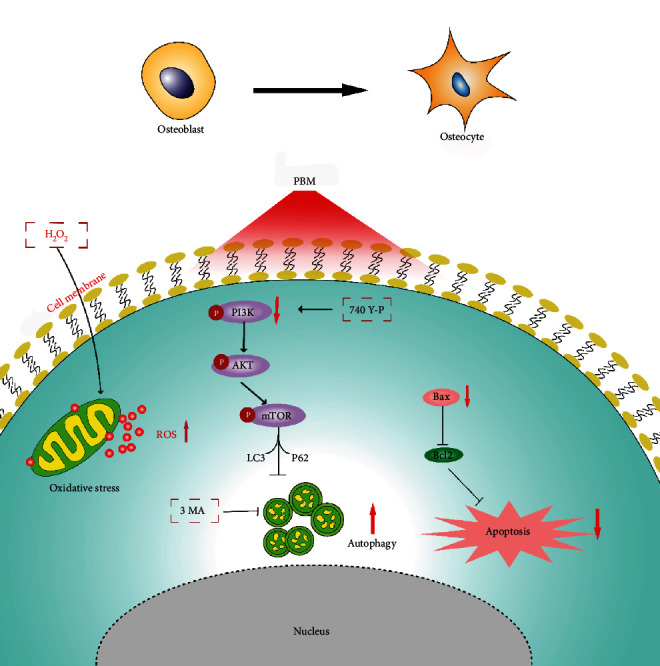
Schematic representation of PBM regulating autophagy in MC3T3 cells during oxidative stress via PI3K/AKT/mTOR pathway.

In brief, we cultured MC3T3-E1 osteoblast precursor cell with an appropriate concentration of H_2_O_2_, constructed an osteoblast oxidative stress model, and irradiated the cells with near infrared light at 808 nm. We observed that photobiomodulation attenuated H_2_O_2_-induced oxidative stress in MC3T3-E1 cells and activated autophagic osteoblasts in response to oxidative stress. In addition, we found that PBM enhanced autophagy by inhibiting PI3K/AKT/mTOR pathway-related signaling proteins, thereby promoting osteoblast differentiation (Figure 7). These findings suggested that photobiomodulation has a beneficial impact on H_2_O_2_-induced osteogenic differentiation of MC3T3-E1 cells.

## Figures and Tables

**Table 1:** Radiant parameters.

	Parameter	Units
Wavelength	808	nm
Spectral bandwidth	5	nm
Wave type	Continuous wave	/
Frequency	50	kHz
Power density	6	mW/cm^2^
Irradiation time	630	s
Energy density	3.78	J/cm^2^
Irradiation frequency	24	h
Distance of irradiation to bottom of plate	21	nm
Irradiated energy	17.01	J

**Table 2:** Primer sequences used in RT-quantitative PCR.

Genes	Forward primer sequence (5′–3′)	Reverse primer sequence (5′-3′)
RUNX2	GAACCAAGAAGGCACAGACAGA	GGCGGGACACCTACTCTCATAC
Osterix	GATGGCGTCCTCTCTGCTT	TATGGCTTCTTTGTGCCTCC
Osteocalcin	ACCATCTTTCTGCTCACTCTGCT	CCTTATTGCCCTCCTGCTTG
Osteopontin	TACGACCATGAGATTGGCAGTGA	TATAGGATCTGGGTGCAGGCTGTAA
*β*-Actin	CATCCGTAAAGACCTCTATGCCAAC	ATGGAGCCACCGATCCACA

## Data Availability

The raw data supporting the conclusions of this article will be made available by the authors, without undue reservation, to any qualified researcher.

## Supplementary Materials

Supplementary Materials Figure S1: the effect of PBM on H_2_O_2_-induced Bcl-xl and bad expression in MC3T3 cells was detected by western blot. Bad expression level was significantly upregulated, and Bcl-xl protein expression level was downregulated after H_2_O_2_ treatment in MC3T3 cells. After irradiation with PBM, enhanced Bcl-xl expression and decreased expression were observed. Figure S2: PBM can promote the expression of autophagy protein Beclin-1 induced by H_2_O_2_ in MC3T3 cells. Figure S3: autophagy inhibitor reversed the effect of PBM on the expression of Beclin-1 in H_2_O_2_-induced MC3T3 cells, and the presence of 3-MA significantly promoted H_2_O_2_-induced MC3T3 cell apoptosis.
