# Supplementary material for: Protective Effect of Photobiomodulation against Hydrogen Peroxide-Induced Oxidative Damage by Promoting Autophagy through Inhibition of PI3K/AKT/mTOR Pathway in MC3T3-E1 Cells
Source: Oxid Med Cell Longev. 2022 Nov 22;2022:7223353. doi: 10.1155/2022/7223353 (PMC9708376; doi:10.1155/2022/7223353)
Supplement: Supplementary Materials — Figure S1: the effect of PBM on H2O2-induced Bcl-xl and bad expression in MC3T3 cells was detected by western blot. Bad expression level was significantly upregulated, and Bcl-xl protein expression level was downregulated after H2O2 treatment in MC3T3 cells. After irradiation with PBM, enhanced Bcl-xl expression and decreased expression were observed. Figure S2: PBM can promote the expression of autophagy protein Beclin-1 induced by H2O2 in MC3T3 cells. Figure S3: autophagy inhibitor reversed the effect of PBM on the expression of Beclin-1 in H2O2-induced MC3T3 cells, and the presence of 3-MA significantly promoted H2O2-induced MC3T3 cell apoptosis. [file 7223353.f1.docx]

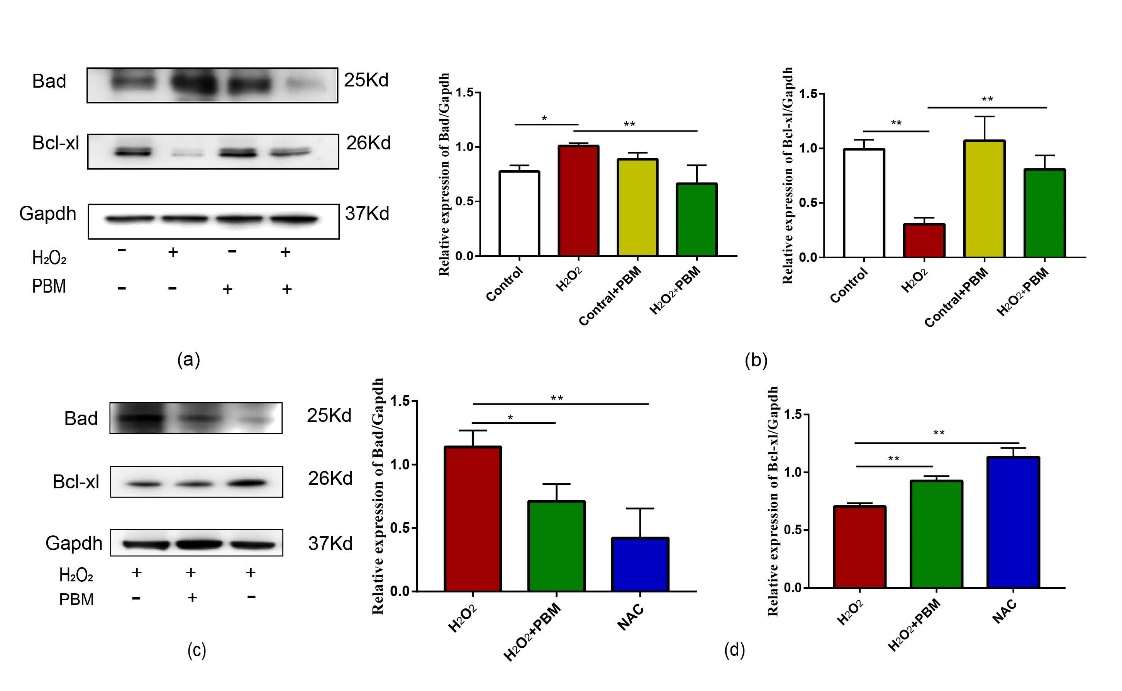


Supplementary figure 1. PBM inhibited H_2_O_2_-induced apoptosis of MC3T3 cells. (a-b) Western blotting analysis and quantification of the expression levels of Bad and Bcl-xl in Control, H_2_O_2_ and treat with PBM groups (n= 3 individuals per group). (c-d) Western blotting analysis and quantification of the expression levels of Bad and Bcl-xl in H_2_O_2_, treat with PBM group and NAC group (n= 3 individuals per group). **p* < 0.05, ***p* < 0.01.


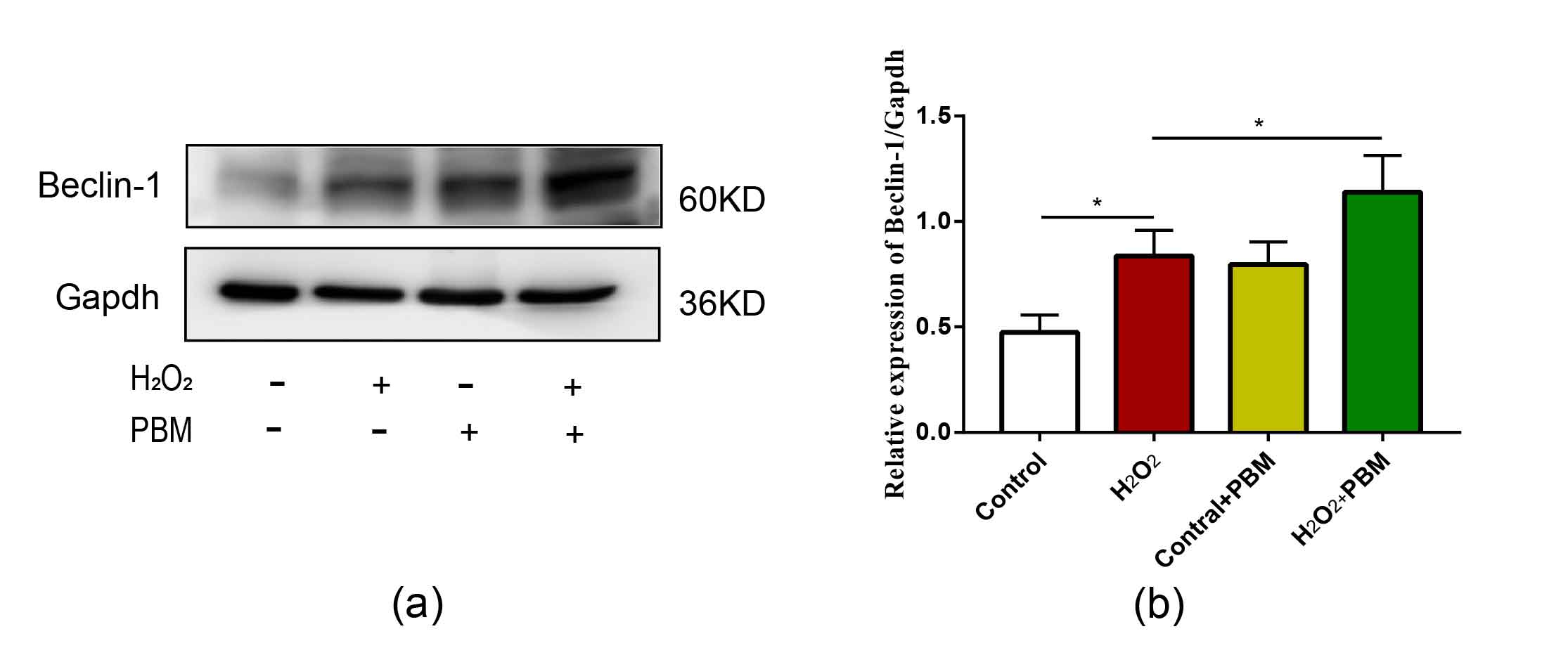


Supplementary figure 2. PBM enhanced the autophagy level induced by H_2_O_2_ in MC3T3 cells. (a-b) Western blotting analysis and quantification of the expression levels of Beclin-1 in Control, H_2_O_2_ and treat with PBM groups (n=3 individuals per group). **p* < 0.05.


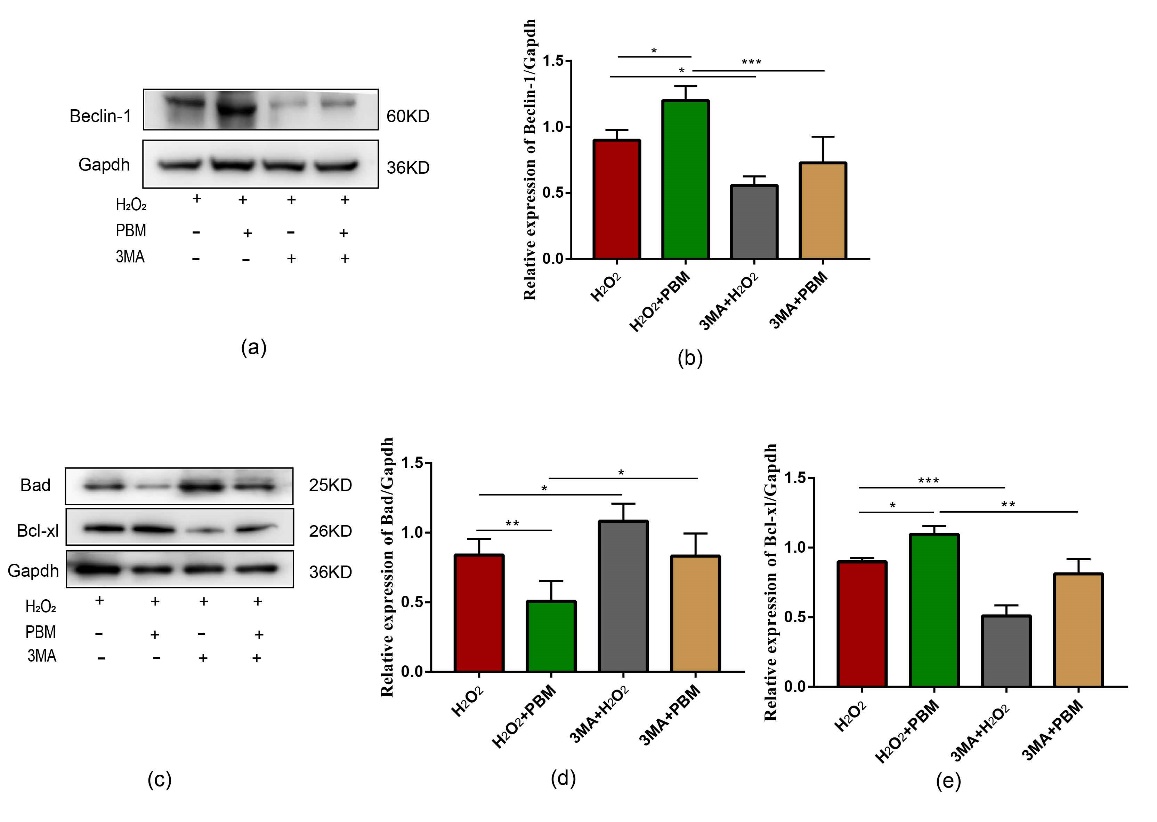


Supplementary figure 3. Autophagy inhibitors can reverse the effect of PBM on H_2_O_2_-induced MC3T3 cells. (a-b) Western blotting analysis and quantification of the expression levels of Beclin-1 in H_2_O_2_, 3MA and treat with PBM groups (n= 3 individuals per group). (c-e) Western blotting analysis and quantification of the expression levels of Bad and Bcl-xl in H_2_O_2_, 3MA and treat with PBM groups (n= 3 individuals per group). **p* < 0.05, ***p* < 0.01, ****p* < 0.001.
